# Genome-Wide Association Study of Resistance to *Phytophthora capsici* in the Pepper (*Capsicum* spp.) Collection

**DOI:** 10.3389/fpls.2022.902464

**Published:** 2022-05-20

**Authors:** Nayoung Ro, Mesfin Haile, Onsook Hur, Bora Geum, Juhee Rhee, Aejin Hwang, Bitsam Kim, Jeaeun Lee, Bum-Soo Hahn, Jundae Lee, Byoung-Cheorl Kang

**Affiliations:** ^1^National Agrobiodiversity Center, National Institute of Agricultural Sciences, Rural Development Administration, Jeonju, South Korea; ^2^Department of Plant Science and Plant Genomics and Breeding Institute, Seoul National University, Seoul, South Korea; ^3^Department of Horticulture, Chonbuk National University, Jeonju, South Korea

**Keywords:** disease resistance, GWAS, HRM marker, pepper germplasm, Phytophthora blight, SNPs

## Abstract

One of the most serious pepper diseases is Phytophthora blight, which is caused by *Phytophthora capsici.* It is crucial to assess the resistance of pepper genetic resources to Phytophthora blight, understand the genetic resistances, and develop markers for selecting resistant pepper materials in breeding programs. In this study, the resistance of 342 pepper accessions to *P. capsici* was evaluated. The disease severity score method was used to evaluate the phenotypic responses of pepper accessions inoculated with the KCP7 isolate. A genome-wide association study (GWAS) was performed to identify single nucleotide polymorphisms (SNPs) linked to *P. capsici* (isolate KCP7) resistance. The pepper population was genotyped using the genotype-by-sequencing (GBS) method, and 45,481 SNPs were obtained. A GWAS analysis was performed using resistance evaluation data and SNP markers. Significantly associated SNPs for *P. capsici* resistance at 4 weeks after inoculation of the GWAS pepper population were selected. These SNPs for Phytophthora blight resistance were found on all chromosomes except Chr.05, Chr.09, and Chr.11. One of the SNPs found on Chr.02 was converted into a high-resolution melting (HRM) marker, and another marker (QTL5-1) from the previous study was applied to pepper accessions and breeding lines for validation and comparison. This SNP marker was selected because the resistance phenotype and the HRM marker genotype matched well. The selected SNP was named Chr02-1126 and was located at 112 Mb on Chr.02. The Chr02-1126 marker predicted *P. capsici* resistance with 78.5% accuracy, while the QTL5-1 marker predicted resistance with 80.2% accuracy. Along with the marker for major quantitative traits loci (QTLs) on Chr.05, this Chr02-1126 marker could be used to accurately predict Phytophthora blight resistance in pepper genetic resources. Therefore, this study will assist in the selection of resistant pepper plants in order to breed new phytophthora blight-resistant varieties.

## Introduction

Pepper is a valuable vegetable crop that is grown for both fresh and processed purposes worldwide. The soil-borne oomycete plant pathogen *Phytophthora capsici* is the most destructive disease, causing up to 100% yield loss in hot (25–28°C) and humid environmental conditions (Foster and Hausbeck, 2010). *Phytophthora capsici* infect pepper plants at almost all stages of growth and development. In the open field, the disease starts to occur after planting, then increases rapidly after the rainy season and continues until mid-September in Korea.

Phytophthora blight is a polycyclic disease, meaning that the pathogen re-infects crops several times under favorable conditions throughout the growing season. *Phytophthora capsici* has two mating types (A1 and A2) that are morphologically identical but genetically distinct (Hausbeck and Lamour, 2004). When both mating types are present in one field, they mate to produce survival structures called oospores. The oospores can live for years in the soil and serve as the initial inoculum for disease development in the spring when conditions are conducive. When contaminated soils are saturated for several hours and temperatures are relatively warm, *P. capsici* will form structures called sporangia, which contain asexual, swimming zoospores that are released into the saturated soil. Zoospores are attracted to living plant parts in the soil and on the soil surface and swim toward them. Once they find a host plant, zoospores can germinate and infect any plant part, either in the soil (roots and crowns) or *via* splashing water (leaves and fruit; Hausbeck and Lamour, 2004).

Several landraces of pepper have been identified to contain resistance genes to *P. capsici* (Kimble and Grogan, 1960; Candole et al., 2010). Exploiting these resources in practical breeding is the most environmentally friendly and promising approach to managing this destructive pathogen compared to chemical and fungicide controls (Barchenger et al., 2018). The pepper line Criollo de Morales 334 (CM334), a landrace with small Serrano-type fruit, has been demonstrated to have resistance to a variety of *P. capsici* isolates from different hosts and geographic regions (Glosier et al., 2008). The inheritance of resistance in CM334 is not completely understood. In Korea, breeding materials, such as AC2258, CM334, and PI 201234, have been introduced since 2005, and varieties resistant to *P. capsici* have been released. To date, many *P. capsici*-resistant varieties derived from CM334 are on the market in Korea (Jo et al., 2014).

The use of molecular markers in plant breeding has led to marker-assisted breeding, which has increased the probability of selection of potential crops and reduced the cost and time for cultivar development compared with conventional breeding techniques (Xu and Crouch, 2008). Among the marker types, single nucleotide polymorphisms (SNPs) are the most abundant source of polymorphisms derived from single base changes in homologous DNA fragments, and they are quickly and easily detected with various techniques (Kim and Misra, 2007; Mammadov et al., 2012). Recently, high-resolution melting (HRM) and Kompetitive allele-specific PCR (KASP) methods have been most widely used for SNP genotyping. HRM is a PCR-based method to detect the difference in melting temperatures resulting from SNPs in amplicons and can be quickly and inexpensively performed because there is no electrophoresis step (Simko, 2016). Phytophthora blight resistance in pepper shows polygenetic inheritance and higher order-epistasis effects (Truong et al., 2012; Curtis, 2014). Several studies have been conducted to find quantitative trait loci (QTL) associated with *P. capsici* resistance and to transfer these QTLs into elite materials (Liu et al., 2014). The late blight resistance of peppers is recognized as QTL resistance, in which six regions present on chromosomes 4, 5, 6, 11, and 12 are responsible for resistance (Siddique et al., 2019). It has been reported that *P. capsici* resistance genes are clustered on chromosome 5 (Siddique et al., 2019). On other chromosomes, QTLs with epistatic interactions and minor effects that were distinct to the isolate and environment were found. Although several molecular markers linked to *P. capsici* resistance in pepper have been identified (Liu et al., 2014; Wang et al., 2016; Xu et al., 2016; Siddique et al., 2019), these molecular markers are not widely applicable for breeding, and some levels of phenotype and genotype mismatches have been observed when applied to diverse germplasm.

It is critical to identify resistance genetic resources and develop an efficient screening approach for a large number of germplasm resources in order to breed novel varieties resistant to Phytophthora blight. Phytophthora blight resistance evaluation with KCP7 isolate in the genome-wide association study (GWAS) of the pepper population was performed using GBS and GWAS analysis. The objectives of this study were to screen pepper genetic resources to *P. capsici* resistance, understanding the genetic resistance of pepper, and develop markers to assist breeding programs in selection of resistant pepper materials. As a result, we identified resistant pepper materials to *P. capsici* that can be used to breed new varieties and SNPs associated with *P. capsici* resistance were identified. A marker (Chr02-1126) was developed to select resistant pepper materials to *P. capsici* and its prediction accuracy (78.5%) was validated. Therefore, the marker developed in this study can be used with other markers to improve the selection accuracy in pepper breeding.

## Materials and Methods

### Plant Materials

In the current study, the pepper population (Lee et al., 2016) was tested for resistance to Phytophthora blight using GWAS. The passport data and the geographical origin of pepper accessions were provided by the National Agrobiodiversity Center (NAC). Ten to twelve plants were planted in a greenhouse at NAC (Jeonju, Korea), and their genetic uniformity and fruit characteristics were evaluated for each accession. Five commercial varieties and 20 breeding lines (Hana Seed Co., Ltd., Korea) were used for marker analysis. *Capsicum annuum* cv. “Manitta” (Nongwoo bio Co., Ltd., Korea) was used as the susceptible control, and the resistant controls were *C. annuum* cv. “Dokyachungchung” (Syngenta Korea Co., Ltd., Korea), *C. annuum* cv. “Bigstar” (Nongwoo bio Co., Ltd., Korea), and *C. annuum* cv. “CM334.”

### Assessment of Phytophthora Blight Resistance

The evaluation of resistance to *P. capsici* KPC-7 in the pepper population was performed at the Rural Development Administration (RDA; Jeonju, Republic of Korea) in 2018. *Phytophthora capsici* KPC-7 isolate was generously provided by Gyung Ja Choi (Dr.) for this study (Korea Research Institute of Chemical Technology, KRICT). Jo et al. (2014) reported that KPC-7 isolate have high virulence. A preliminary experiment was conducted to confirm the pathogenicity of *P. capsici* isolate, and isolate was re-separated from damaged plant tissues before being used for inoculation. In order to inoculate pepper accessions, isolate was cultured at 25°C under fluorescent light illumination on potato dextrose agar (PDA) medium for mycelium growth until the plates were completely covered in mycelia.

To harvest mycelium, the PDA plates with mycelium were washed with distilled water. Prior to inoculation, the spore concentration was measured using a hemocytometer, and the spore suspension density was adjusted to around 5 × 10^4^ sporangia/ml. To encourage zoospore release, the sporangial suspension was cooled at 4°C for 1 to 2 h before inoculation, then transferred to 25°C for 1 h.

**Figure 1:**
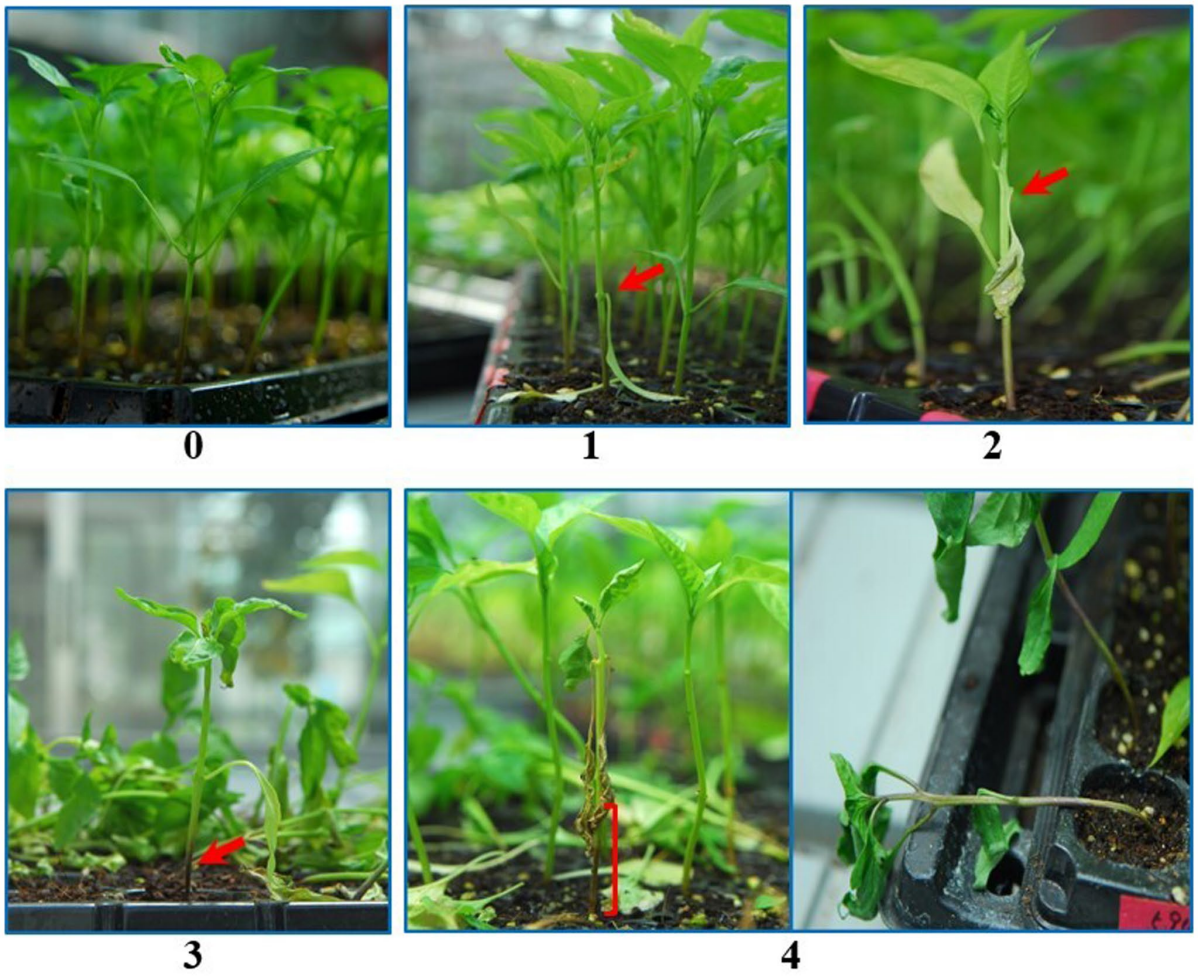
Disease scales (0–4) used to distinguish the resistant and susceptible plants. Pictures were taken at 4 weeks after inoculation. 0 (resistant) = no visible symptoms, 1 (resistant) = dark lesion visible on the base of the stem but surviving without wilting, 2 (moderately resistant) = wilting with a dark lesion at the base of the stem, 3 (susceptible) = wilting with a dark lesion at the medium of the stem, and 4 (highly susceptible) = wilting and death of the whole plant.

A total of 342 pepper core collection accessions were scored for disease resistance with three replications (10 plant/replication) for each accession. The plants were cultivated in 50 cell plastic trays and inoculated with a 5 ml spore suspension drenched at the base of the stem by a dispenser at the 4–6 true leaf stage. The inoculated plants were grown at 25°C in a controlled environment and watered frequently to promote disease establishment. As resistant controls, *C. annuum* cv. CM334, and Dokyachunhchung were used, while *C. annuum* cv. Manitta was used as a susceptible control to compare the resistance levels. The final disease severity was scored at 4 weeks of inoculation based on the disease scale 0–4, where 0 (resistant) = no visible symptoms were observed; 1 (resistant) = a dark lesion visible on the base of the stem, but surviving without wilting; 2 (moderately resistant) = wilting with a dark lesion at the base of the stem; 3 (susceptible) = wilting with a black lesion on the stem’s middle; and 4 (highly susceptible) = wilting and death of the entire plant as described by Mo et al. (2014) (Figure 1).

We used 96 pepper accessions and varieties that had previously been studied for phytophthora disease resistance to validate markers. Among the 96 pepper materials, 64 were resistant (disease score, 0–1) and 32 were susceptible (disease score; 3.1–4). The markers were also validated with 20 pepper breeding lines from Hana Seed Co., Korea.

### Extraction of gDNA and Genotyping-by-Sequencing

With the CTAB method, genomic DNA was extracted (Lee et al., 2016) and diluted to 50 ng/μl using distilled water. DNA was quantitated using the standard procedure of the Quant-iT PicoGreen dsDNA Assay Kit (Molecular Probes, Eugene, OR, United States) with the Synergy HTX Multi-Mode Reader (Biotek, Winooski, VT, United States) and standardized to 12.5 ng/μl. DNA was digested with ApeKI (New England Biolabs) at 75°C for 3 h.

The genotyping-by-sequencing (GBS) libraries were built using the procedures described previously (Elshire et al., 2011; De Donato et al., 2013), with minor changes. After restriction digestion, the DNA was ligated with adapters. Adapters with barcodes to identify individual samples and common adapters were among the adapters. T4 DNA ligase (New England Biolabs) was used to ligate the DNA at 22°C for 2 h, after which the ligase was inactivated by keeping it at 65°C for 20 min. The adapter ligated samples were combined into a single sample and purified with the NucleoSpin® Gel and PCR Clean-up Kit (MACHEREY-NAGEL GmbH & Co. KG). Multiplexing PCR was used to amplify the pooled ligations in a 50-L reaction using AccuPower Pfu PCR Premix (Bioneer) and 25 pmol of each primer listed below; 5′-AATGATACGGCGACCACCGAGATCTACACTCTTTCCCTA CACGACGCTCTTCCGATCT-3′ and 5′-CAAGCAGAAGACG GCATACGAGATCGGTTCGGCATTCCTGCTGAACCGCTCT TCCGATCT-3′

The PCR products were evaluated for the distribution of fragment sizes with the BioAnalyzer 2100, which was used to analyze the PCR products for fragment size distribution (Agilent Technologies). The GBS libraries were sequenced with 150 bp single-end reads on the Illumina NextSeq500 (Illumina, San Diego, CA, United States).

### Sequence Preprocessing and SNP Calling

Sequence preprocessing on produced reads was conducted with Stacks, FastQC, and Cutadapt software. Demultiplexing was performed using Stacks, “process radtags” module. After that, FastQC and Cutadapt were used to perform quality control on per-base read quality and to remove possible adaptor sequences. Bowtie2 was used to align the reads to the CM334 reference genome (*C. annuum* chromosome v1.6; Langmead and Salzberg, 2012). To add read groups to the reads, the command-line Picard tools^1^ were employed, allowing the reads to be used in the GATK pipeline. GATK was used to conduct local read realignments to rectify misalignments caused by indels (“IndelRealigner” and “RealignerTargetCreator” arguments). For calling candidate SNPs aligned to the pepper chromosome v1.6 reference genome, the “HaplotypeCaller” and “SelectVariants” arguments were used. After obtaining raw variants, GATK’s “filterVariant” module was used to filter out variants based on quality score (QUAL < 30), quality depth (QD < 5), Fisher score (FS > 200) and with vcftools v. 0.1.15 to restrict the missing rate (--max-missing 0.95), minor allele frequency (--maf 0.05), a number of alleles (--min-alleles 2, --max-alleles 2), and mean read depth for a SNP locus (--min-meanDP 5).

### Population Structure and Relationships

The VCF file containing filtered SNPs was converted to plink format using PLINK software (Purcell et al., 2007). Principal component analysis (PCA) of genetic variation among five groups was performed using GCTA software (Yang et al., 2011). In order to investigate the population structure of samples, ADMIXTURE (Alexander et al., 2009) was conducted with population ancestry (*K*) values ranging from 2 to 20. Then the delta *K* value was calculated from the likelihood value for each *K* value, and an admixture plot per sample was visualized for the highest delta *K* value. Nucleotide diversities for each of the five groups were performed using VCFtools (Danecek et al., 2011).

### Genome-Wide Association Analysis

The GWAS analysis was carried out using TASSEL v.5.0 standalone. The kinship (K) matrix was computed based on familial relatedness between lines in an identical-by-state (IBS) matrix. Transformations using the R package’s box-cox function were used to fix the skewed problem of trait distribution. Then, using the QK model (Bastien et al., 2014), a mixed linear model (MLM) was fitted with corrections for both population structure and relatedness. The Genome Database (chromosome v1.6)^2^ was used to predict potential genes using the loci of significant SNPs.

### SNP Identification and HRM Primer Design

As a reference genome, *C. annuum* cv. CM334 genomic data (chromosome v1.6) was used. Several raw SNPs were detected using the SAMtools (0.1.16) program, and consensus sequences were generated concurrently (Li and Durbin, 2009). The total SNP matrix between the samples was generated using an in-house script developed by Cho and Kim (Kim et al., 2014). Finally, the detected SNPs’ flanking sequences (400 bp) were aligned to the reference genome using a BLASTN search to identify non-paralogous SNPs. Primers were designed for sequences with only one copy in the reference genome. Primer3web ver. 4.1.0^3^ was used to design a total of 32 primer sets for HRM analysis.

### HRM Analysis

The total reaction volume for HRM analysis was 20 μl, containing 10 ng of genomic DNA, 2.0 μl of 10 × Easy Taq buffer (TransGen Biotech, Beijing, China), 1.0 μl of 2.5 mM dNTP mixture (TransGen Biotech), 0.1 μl of Easy Taq DNA polymerase (Transgen Biotech), 1.0 μl of SYTO®9 green fluorescent nucleic acid stain (Life Technologies™, Carlsbad, CA, United States), 0.5 μl each of 10 pmol μl^−1^ of a pair of primers (Supplementary Table S1), and autoclaved distilled water for the remainder of the volume. On the Biometra TAdvanced (Biometra GmbH, Göttingen, Germany), the following PCR conditions were used: initial denaturation at 95°C for 5 min; denaturation at 95°C for 10 s, followed by annealing and elongation at 60°C for 20 s, repeated 40 times; and final denaturation at 95°C for 10 s. HRM analysis was performed using the LightCycler® 96 Real-Time PCR System (Roche, Basel, Switzerland) at each temperature during a 0.3% rise from 60 to 90°C. The HRM graphs were drawn by LightCycler® 96 software ver. 1.1 (Roche).

## Results

### Screening for *Phytophthora capsici* Resistance in Pepper Collections

The pepper populations, including resistant and susceptible controls, were evaluated for resistance to the *P. capsici* isolate KCP-7 in the growth room. The control varieties used in this study are listed in Supplementary Table S2. The resistant controls showed no disease symptoms (Supplementary Table S2). The susceptible controls, on the other hand, were entirely susceptible (Supplementary Table S2). The disease severity scores of the resistant and susceptible controls showed that the KPC-7 isolate inoculation was successful. The disease severity scores of CM334 and Dokyachungchung were 0, and that of Big Star was 0.2, while the disease severity scores of Manitta, Chungyang, and Jeju Jarae were 4 (Supplementary Table S2). After inoculation with *P. capsici* KPC-7, the pepper accessions showed varying degrees of disease resistance.

**Figure 2:**
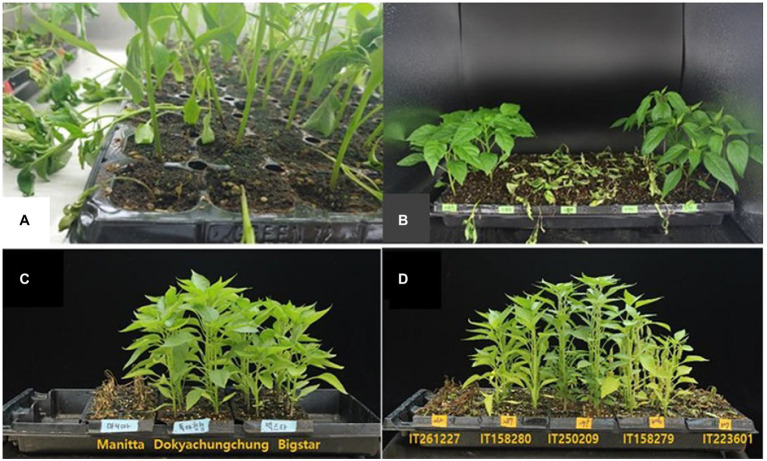
*Phytophthora capsici* symptoms on susceptible and resistant *Capsicum* accessions. Weekly changes in disease development are shown **(A,B)**. After 1 week of inoculation, the stem of the susceptible accessions started to dry and, in severe cases, fall **(A,B)**. **(C,D)** Shows *Phytophthora capsici* symptoms of susceptible and resistant *Capsicum* accessions at 4 weeks after inoculation. **(C)** Control plants: Manitta is susceptible, and Dokyachungchung and Bigstar are resistant. **(D)** Representative core collections: IT261227 and IT223601 are susceptible, and IT158280, IT250209, and IT158279 are resistant.

The weekly changes in disease development are shown in Figure 2. The stems of susceptible accessions began to dry and, in extreme cases, fell after 1 week of inoculation (Figures 2A,B). *Phytophthora capsici* symptoms were observed 4 weeks after the inoculation of susceptible and resistant *Capsicum* accessions (Figures 2C,D). The control plants (Manitta is susceptible, and Dokyachungchung and Bigstar are resistant) are shown in Figure 2C. The representative core collections are presented in Figure 2D. IT261227 and IT223601 were susceptible, whereas IT158280, IT250209, and IT158279 were resistant.

**Figure 3:**
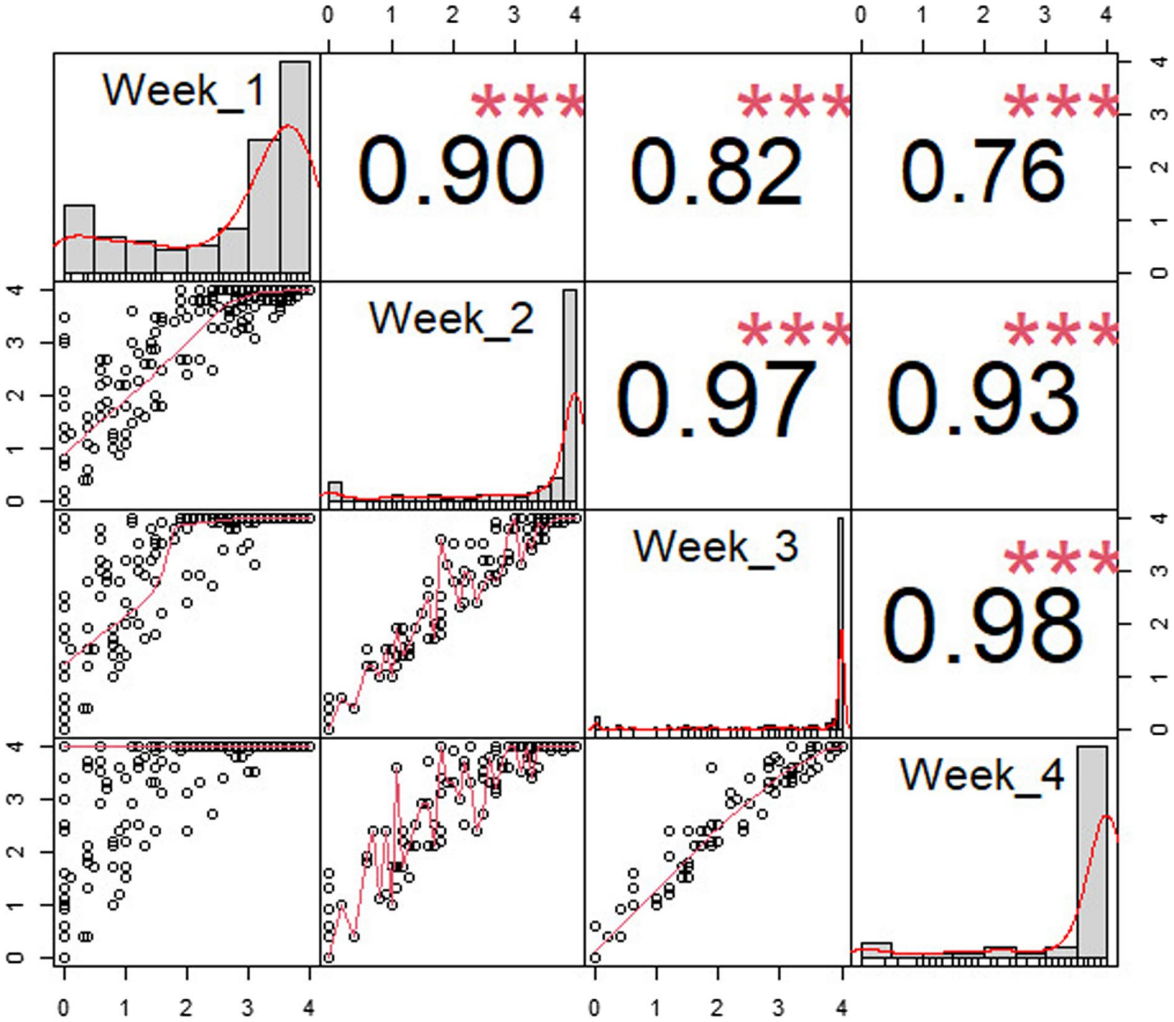
Pearson correlation of disease resistance by weeks after inoculation. X- and Y-axes are disease severity scores, and dots were drawn on the XY plane according to the disease score. The red line represents the relationship between X- and Y-axes, and the histograms represent number individuals of each disease severity score in each week. The sign “***” means: highly significant (*p* < 0.001).

Table 1 depicts the number of accessions showing resistance during the first 4 weeks following *P. capsici* KPC-7 inoculation. The correlation analysis revealed significant positive correlations between *P. capsici* resistances at different weeks after inoculation. The correlation coefficient between 1 week and 4 weeks after inoculation was 0.76, whereas it was 0.98 between 3 and 4 weeks (Figure 3). After 4 weeks, resistant accessions remained healthy, whereas susceptible accessions were completely dead. Four weeks after inoculation, resistant accessions were transferred to pots to observe growth, and most of them flowered and set fruits normally.

**Table 1:** Distribution of resistant plants in the pepper population by weeks after inoculation with KCP7 isolate.

Disease severity score	Number of accessions			
	1 week after inoculation	2 weeks after inoculation	3 weeks after inoculation	4 weeks after inoculation
0–1	61	31	25	22
1.1–2	33	25	22	12
2.1–3	42	26	18	18
3.1–4	206	260	277	290

The disease evaluation of the pepper population according to *Capsicum* spp. is summarized in Table 2. Of *C. annuum*, eight accessions had a severity score ranging from 0 to 1, six had a score ranging from 1 to 2, and 11 had a score ranging from 2 to 3. The remaining 197 accessions had severity levels ranging from 3 to 4. Among the collection, CM334, CM331, and six other accessions were entirely resistant to isolate KPC-7, whereas MC 11, Saceon 94187, and four other accessions were somewhat resistant, with minor disease signs at the stem collars. Two accessions with moderate resistance were also identified: one (PI 439449) from *Capsicum chinense* and another one (PI 82005) from *Capsicum frutescens*. There was no resistant accession in the *Capsicum baccatum* or *Capsicum chacoense* accessions. The disease evaluation results of the eight resistant accessions belonging to *C. annuum* are presented in Table 3.

**Table 2:** Distribution of *Phytophthora capsici* disease severity score at 4 weeks after inoculation of pepper population with KCP7 isolate.

Species	Number of plants				Total
	Disease severity score				
	0–1	1.1–2	2.1–3	3.1–4	
*Capsicum annuum*	8	6	11	197	222
*Capsicum baccatum*				48	48
*Capsicum chacoense*				2	2
*Capsicum chinense*			1	44	45
*Capsicum frutescens*			1	24	25
Total	8	6	13	315	342

**Table 3:** List of selected resistant accessions.

Accession Number	Scientific name	Accession name	Disease severity score
IT158714	*Capsicum annuum*	PANGALENGAN-1	0.6
IT 231151	*Capsicum annuum*	Ibam	0.5
K150013	*Capsicum annuum*	YCM334	0.0
IT 236424	*Capsicum annuum*	Jungangjongmyo-2000-5032	0.8
IT 240016	*Capsicum annuum*	Jalapeno Mucho Nacho	0.5
IT 158280	*Capsicum annuum*	Szechwan 8	0.0
IT 250209	*Capsicum annuum*	CM334	0.5
IT 236422	*Capsicum annuum*	CM331	0.0

### SNPs and Genome-Wide Association Study

**Figure 4:**
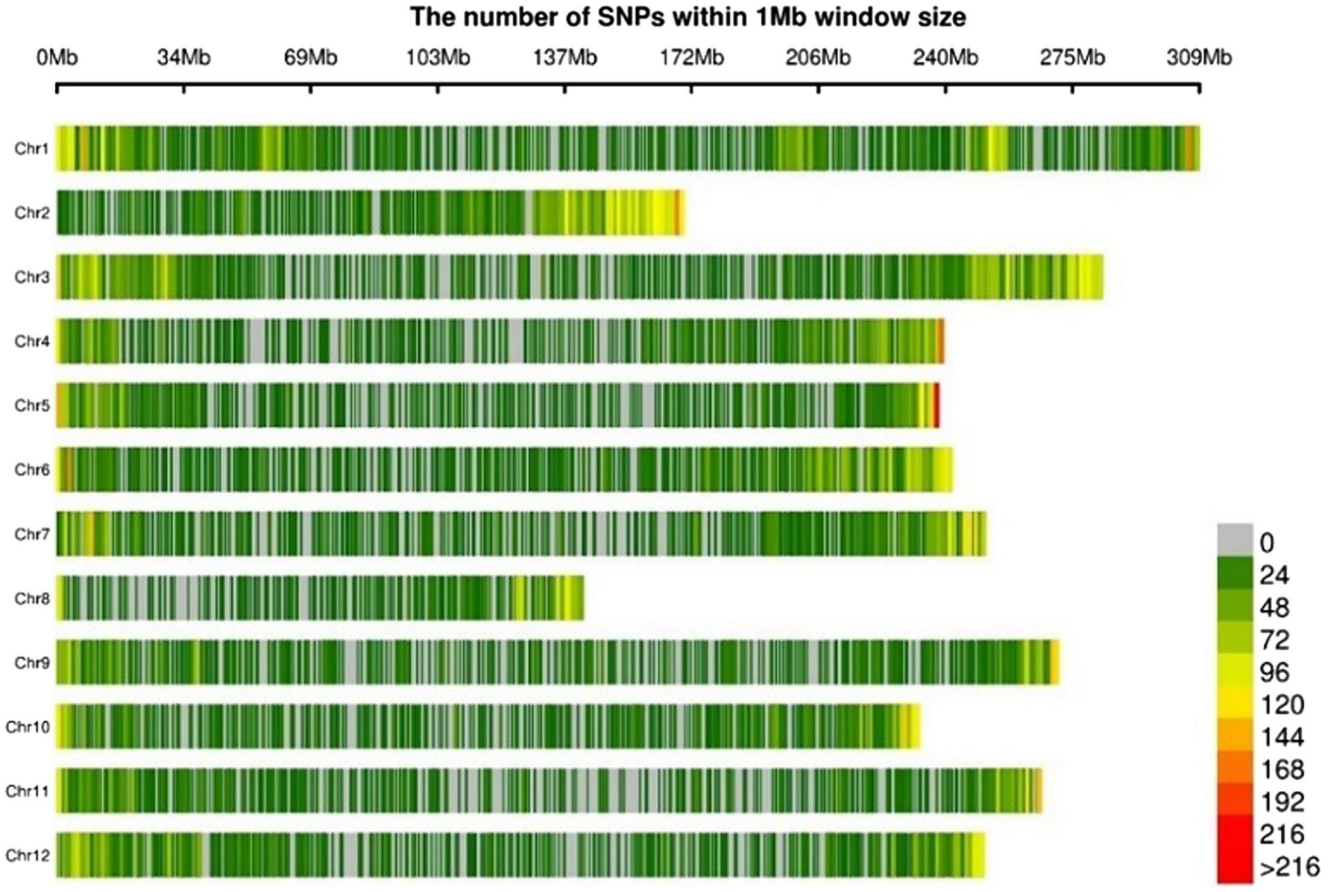
Physical distribution of SNPs (number of SNPs per 1 Mb) derived from genotype-by-sequencing on 12 chromosomes of core collections.

Genotypes of the pepper population were analyzed using GBS after the preparation of libraries of ApeKI-digested gDNA. The average number of reads per sample was around 608 million. The average sequencing depth of each accession was 15.2×. A total of 45,481 SNPs, distributed across all 12 chromosomes, were detected by aligning the sequences obtained from GBS with *C. annuum* cv. CM334 reference genome ver. 1.6 (Kim et al., 2017). The SNPs were more densely distributed at the ends of the chromosomes (Figure 4). The ratio of homo/hetero genotype was calculated for each sample, using the in-house script of Cho & Kim Cor (Supplementary Figure S1). As shown in Figure 4, the GWAS of the pepper population can be considered fairly uniform.

**Figure 5:**
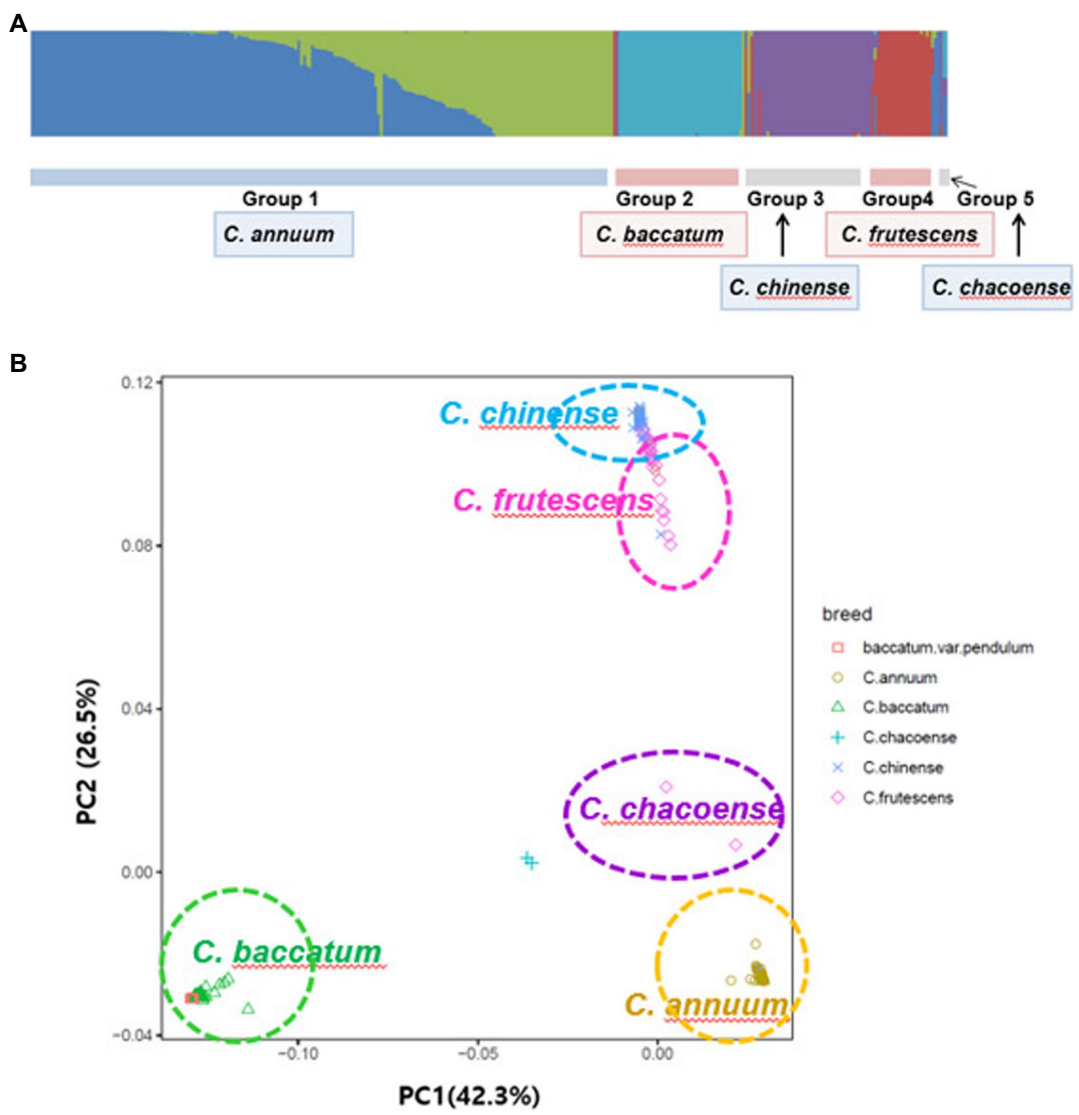
Population structure of the GWAS of pepper population with the fastStructure program **(A)** and a principal component analysis **(B)**.

Each sample used in the phenotype data by the fastStructure program was confirmed to assume the best model when *K* = 5 fastStructure was based on a variational Bayesian framework (Figure 5A). The principal component analysis, PC1 and PC2, explained 42.3 and 26.5% of the variations in the entire sample, respectively (Figure 5B). *C. chinense*, *C. frutescens*, *C. annuum*, and *C. baccatum* each formed a group, and two samples of *C. chacoense* were separated.

**Figure 6:**
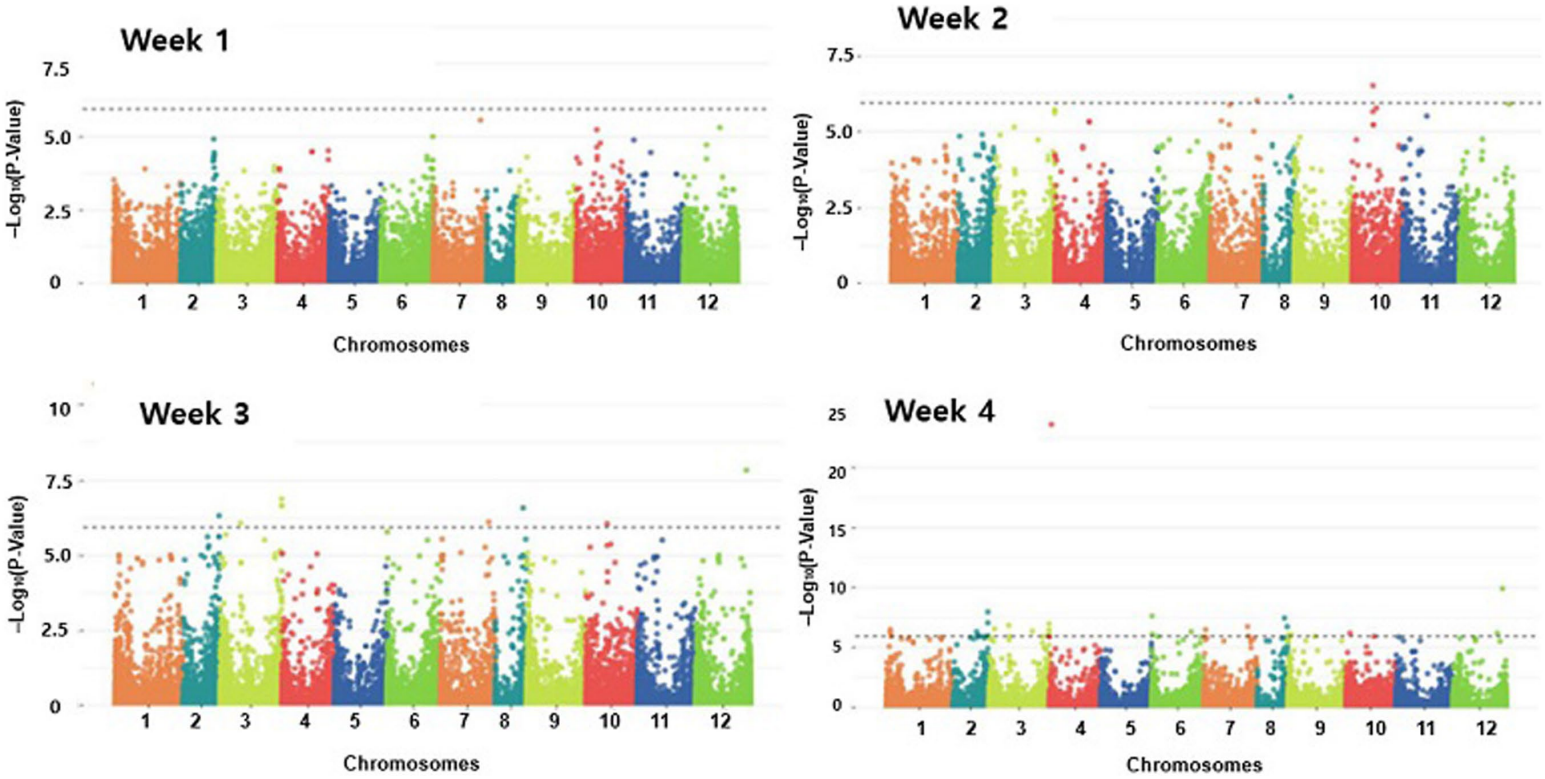
Manhattan plots of genome-wide association study on Phytophthora resistance in GWAS of pepper population. GWAS analysis was performed weekly to show the Manhattan plot.

To perform a GWAS analysis, normality was checked using the Shapiro test (Shapiro and Wilk, 1965). For the inoculation data, a correction was performed on the data that did not satisfy normality. The GWAS analysis was performed using additional information of kinship and structure using the Tassel program and the fastStructure program. The model for the association study was tested, and the most suitable models for the Manhattan plot and the QQ plot are shown in Figure 6 and Supplementary Figure S2, respectively. The GWAS analysis for resistance results in the pepper population was performed weekly to show the Manhattan plot and the QQ plot. As shown by the Manhattan plots in Figure 6, there were no significant SNPs (with a *p*-value of more than 10) in the first week model. Three SNPs on Chr.07, Chr.08, and Chr.10 chromosomes in the second week model; nine SNPs in the third week model (Chr.2, Chr.03, Chr.07, Chr.08, Chr.09, Chr.10, and Chr.12 chromosomes); and 29 SNPs in the fourth week model (Chr.01, Chr.02, Chr.03, Chr.04, Chr.06, Chr.07, Chr.08, Chr.09, Chr.10, and Chr.12 chromosomes).

### Identification and Selection of SNPs for HRM Markers

Following the Bonferroni correction, significant SNP markers from the association study were extracted. The sequence of *C. annuum* cv. CM334 (chromosome v1.6)^2^ was used as a reference sequence. At 4 weeks after inoculation, the significant SNPs above the threshold −log10 *p*-values associated with resistance to *P. capsici* isolate KPC-7 in the pepper population were selected. The selected SNPs for each chromosome are as follows: Chr.01 was 3, Chr.02 was 6, Chr.03 was 7, Chr.04 and Chr.10 were 1 separately, Chr.06 was 3, and Chr.07, Chr.08, Chr.09, and Chr. 12 were 2 separately (Table 4).

**Table 4:** Significant SNPs associated with *Phytophthora capsici* KPC-7 resistance in the pepper population.

Chr.	Position (bp)	*p*-Value	Ref.^*^	Alt.^*^	Type	Gene name/symbol	Function	Primer design
chr01	18,309,131	3.12E-07	T	A	missense_variant	LOC107869375	Protein NRT1/PTR FAMILY 7.3-like	No
chr01	18,541,809	5.90E-07	T	A	intergenic_region	–	–	No
chr01	18,541,871	5.37E-07	C	G	intergenic_region	–	–	No
chr02	112,632,207	4.85E-07	T	C	upstream_gene_variant	LOC107858831	Protein WVD2-like 7	Yes
chr02	115,557,669	8.66E-07	G	A	intergenic_region	–	–	Yes
chr02	118,473,950	8.83E-07	A	C	downstream_gene_variant	LOC107858923	Uncharacterized	Yes
chr02	165,074,534	8.84E-08	C	A	synonymous_variant	LOC107860606	Peroxidase 63 mRNA	Yes
chr02	165,083,237	1.09E-06	C	A	upstream_gene_variant	LOC107860605	U-box domain-containing protein 40	Yes
chr02	165,083,291	1.10E-08	T	C	upstream_gene_variant	LOC107860605	U-box domain-containing protein 40	Yes
chr03	26,843,710	2.82E-07	G	T	synonymous_variant	LOC107864116	Protein TIME FOR COFFEE	Yes
chr03	92,882,787	1.46E-07	G	C	intergenic_region		–	No
chr03	203,119,513	4.45E-07	T	C	upstream_gene_variant	LOC107863456	luc7-like protein 3	Yes
chr03	273,929,454	1.06E-06	A	G	intron_variant		Signal recognition particle 54 kDa	Yes
chr03	281,084,163	1.07E-07	A	T	synonymous_variant	LOC107864287	F-box/Kelch-repeat protein At3g06240-like	No
chr03	281,374,819	3.75E-07	T	C	intergenic_region	–	–	Yes
chr03	281,374,880	3.77E-07	T	G	intergenic_region	–	–	Yes
chr04	9,578,255	2.47E-24	G	A	intergenic_region	–	–	Yes
chr06	1,385,184	2.39E-08	A	T	intergenic_region	–	–	No
chr06	3,204,310	7.86E-07	G	A	intergenic_region	–	–	Yes
chr06	184,471,306	4.83E-07	G	A	missense_variant	LOC107873008	Uncharacterized	Yes
chr07	9,144,225	3.23E-07	A	G	intergenic_region	–	–	Yes
chr07	205,297,202	1.85E-07	C	T	downstream_gene_variant	LOC107879742	Pentatricopeptide repeat-containing	Yes
chr08	126,466,421	3.57E-08	T	A	downstream_gene_variant	LOC107879742	Hevamine-A-like mRNA	No
chr08	138,930,210	1.86E-07	C	A	upstream_gene_variant	LOC107839408	Phosphomannomutase	Yes
chr09	3,642,467	8.34E-07	T	C	downstream_gene_variant	LOC107843160	Uncharacterized	Yes
chr09	16,370,729	9.33E-07	C	T	missense_variant	LOC107842944	Phosphatidate phosphatase PAH1	Yes
chr10	19,168,172	6.50E-07	C	G	intergenic_region	–	–	No
chr12	208,357,955	6.52E-07	T	A	downstream_gene_variant	LOC107851575	Endoglucanase 6 mRNA	No
chr12	232,050,702	1.24E-10	T	C	downstream_gene_variant	LOC107851266	Succinate dehydrogenase subunit 5	Yes

Chr., Chromosome; Ref., Reference sequence; Alt., Alternate sequence. *p < 0.05.

**Figure 7:**
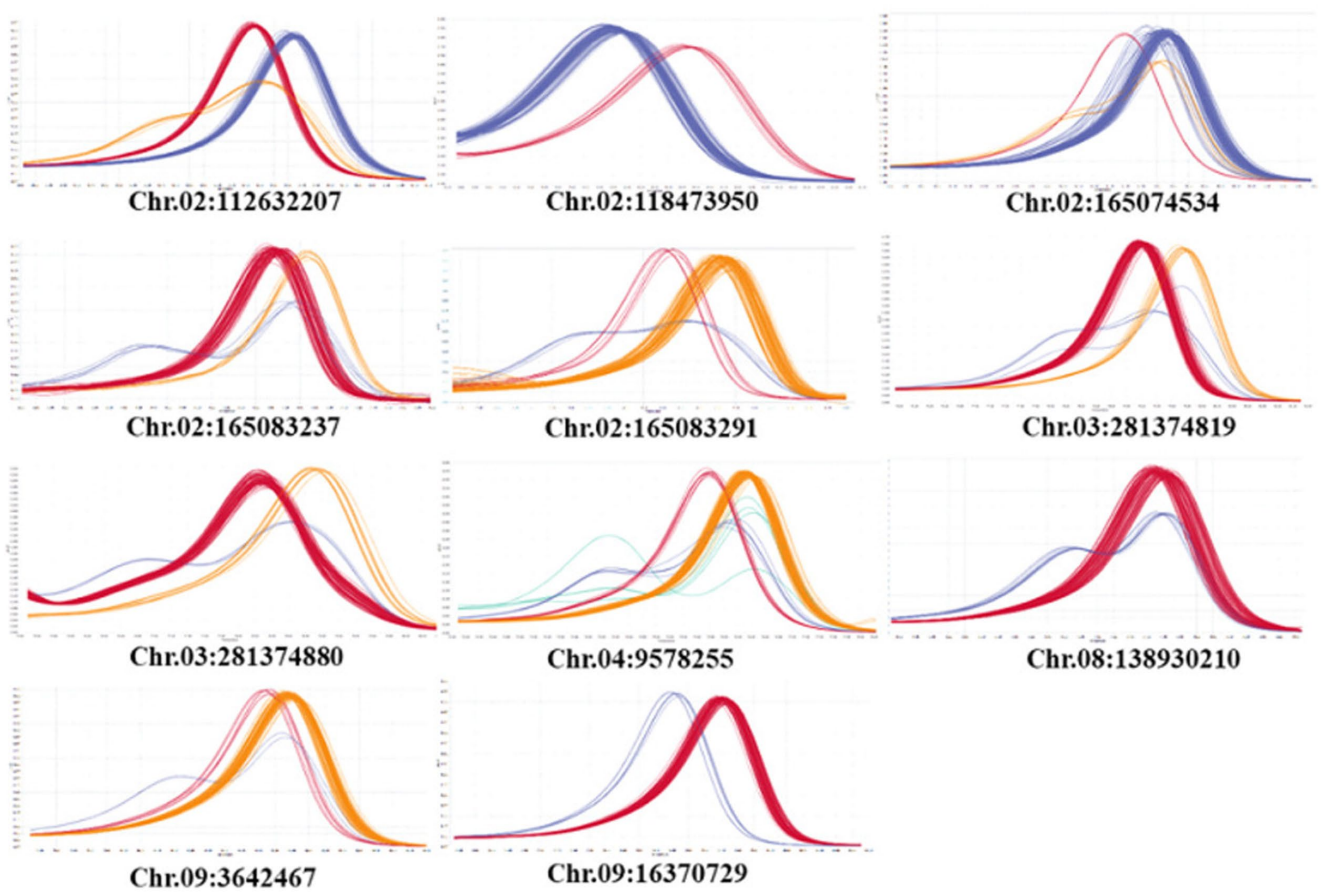
Normalized melting peaks of 11 HRM markers derived from significant SNPs in GWAS analysis of Phytophthora resistance.

**Figure 8:**
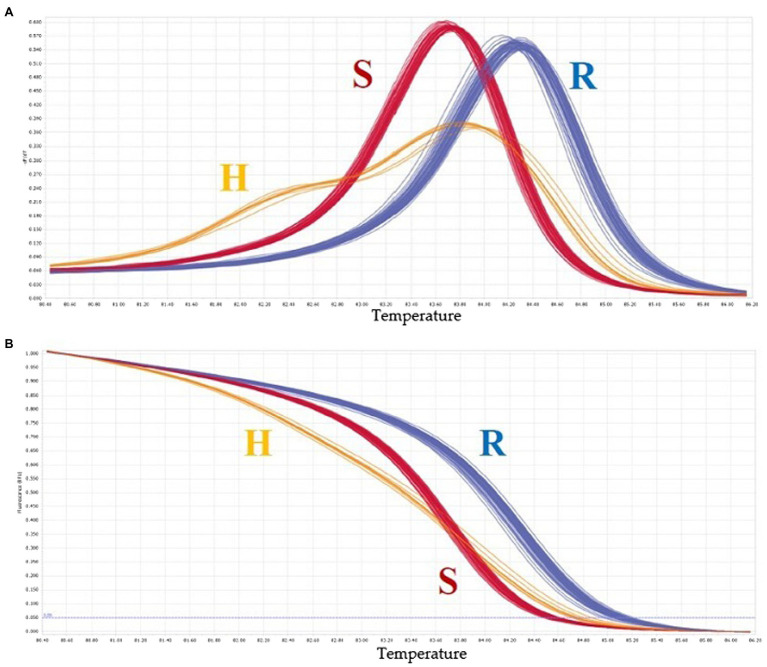
Normalized melting peaks **(A)** and melting curves **(B)** of “Chr02-1126” HRM marker. R, resistant, H, heterozygous, S, susceptible. Chr02-1126 was selected because the resistance phenotype and the HRM marker genotype were matched well.

A total of 20 HRM markers were designed. Excluding the AT and GC mutations, which cannot be analyzed by HRM due to the same number of hydrogen bonds, primers with about 20 base pairs of SNPs were designed so that the product size was 51–150 base, and the TM value was around 60 degrees. Eleven SNPs were polymorphic, eight SNPs were monomeric, and one melting curve pattern was ambiguous in the HRM analysis (Figure 7). Among the 11 polymorphic SNPs, an SNP marker was selected because the resistance phenotype and the HRM marker genotype matched well. The selected SNP was named Chr02-1126. Figure 8 shows the normalized melting peaks (a) and melting curves (b) generated by the Chr02-1126 HRM marker. This HRM marker could clearly distinguish between homozygous resistant, susceptible, and heterozygous genotypes.

### Chr02-1126 HRM Marker Validation

**Figure 9:**
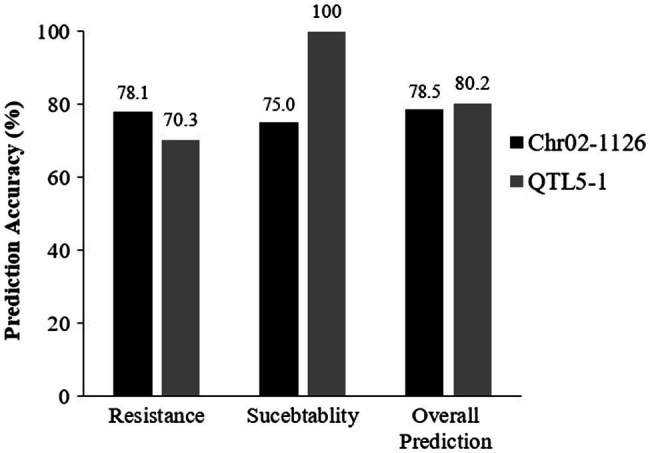
Prediction of Phytophthora resistance of validated populations with Chr02-1126 and QTL5-1 markers. The numbers above the bars represent the percentage of prediction accuracy of Chr02-1126 and QTL5-1. This graph made using the data presented in Table 5.

The Chr02-1126 HRM marker was applied to 96 accessions and varieties to validate its effectiveness in selecting *P. capsici* resistance pepper genetic resources. These samples included 64 *P. capsici*-resistant and 32 susceptible plants that were previously selected from NAC germplasm collections. For validation, we used two markers: Chr02-1126 (112 Mb) from this study and QTL5-1 (18.7–19.5 Mb) from a previous study (Siddique et al., 2019). The marker analysis results of Chr02-1126 for the 96 selected samples are presented in Table 5. When Chr02-1126 SNP was applied to the 96 samples, the phenotype prediction accuracy was about 78.5% (Figure 9). Of the 64 resistant plants, 48 were judged to have homozygous resistance (R) or heterozygous alleles (H), and 13 were judged to have the susceptibility allele (S). Of the 32 susceptible plants, seven were found to have homozygous resistance or heterozygous alleles, and the remaining 25 were determined to have susceptibility alleles. When the QTL5-1 marker was applied, the phenotype prediction accuracy was about 80.2% (Figure 9). Of the 64 resistant plants, 45 were judged to have homozygous resistance or heterozygous alleles, and 19 were judged to have a heterozygous genotype. All 32 susceptible plants were determined to have the homozygous susceptibility allele.

**Table 5:** Comparison of Chr02-1126 and QTL-5 markers for 96 samples in *Capsicum annuum*.

IT	Accession name	Disease severity score	Chr02-1126	QTL5-1
Control-R	Arittaun	R(1.5)	H	H
Control-R	CM334	R(0)	R	R
Control-R	Dokyachungchung	R(0)	H	H
Control-R	Colorstop	R(1)	H	H
Control-R	Bigstar	R(0)	H	H
267516	11PC 3	R(0)	S	R
286568	GN 13042	R(0)	R	R
294689	California Wonder PS	R(0.1)	S	R
203498	Jalapeno Chilli	R(0)	S	R
218765	S.N.KYE1 se	R(0)	R	R
219880	CHAHUA	R(0)	S	R
221878	AC 2258	R(0.9)	R	R
221879	SCM 334	R(0.9)	R	R
223679	KC 00006	R(0.4)	S	R
223789	Corne ll12	R(0.4)	R	R
158279	MC5	R(1)	R	R
270403	250-2-S-1	R(1)	R	R
270404	250-2-S-2	R(1)	R	R
270509	Grif 9109-1	R(1)	R	R
270565	12G195	R(1)	H	R
270566	12G196	R(1)	R	R
270631	PI 201237-3	R(1)	S	R
270632	PI 201237-4	R(1)	S	R
270634	PI 224438-1	R(1)	R	R
270646	PI 640588-2	R(0.6)	S	R
270742	YP 12071	R(1)	R	R
286144	YP 03083	R(1)	R	R
286146	YP 03087	R(1)	R	R
286148	YP 03090	R(1)	S	R
286149	YP 03091-1	R(1)	R	R
286150	YP 03092	R(1)	R	R
293917	KC 01808	R(1)	-	R
293921	YCb 76105	R(1)	S	R
311506	PR 2	R(1)	R	R
311535	04H077	R(1)	R	R
311537	04H085	R(1)	R	S
270455	12G161	R(0)	R	S
270511	12G371	R(0.6)	R	S
270512	12G372	R(0.9)	R	S
270518	Hei lamei xiao jian jiao	R(0.8)	R	R
270522	Hu xiang zao hong	R(0.4)	R	S
270541	YP 98288	R(0)	S	R
270542	YP 98291	R(0)	R	H
270543	YP 98294-2	R(0.4)	R	S
270544	YP 98278	R(0)	R	S
270547	YP 98280-1	R(0)	S	H
270548	YP 98281	R(0)	H	H
270550	YP 98285	R(0.8)	R	S
270555	12G201	R(0.4)	R	S
270556	12G202	R(0.4)	R	S
270557	12G203	R(0)	H	S
270558	12G204	R(0.5)	R	S
270701	Xian you 11	R(0)	R	H
286167	NuMex vaquero	R(1)	S	R
286169	Laail	R(0.8)	R	S
286569	KC00396	R(0.3)	R	S
32379	Gimjang(10)pepper	R(1)	R	S
32385	Bird pepper	R(0.8)	R	S
158280	Szechwan 8	R(0.8)	R	R
158708	C00559	R(0.7)	R	S
158714	PANGALENGAN-1	R(0.5)	H	H
158720	C00595	R(0.6)	R	S
223737	VP 55	R(0.3)	–	S
223783	MC 11	R(1)	–	R
Control-S	Manitta	S(4)	S	S
Control-S	Jeju-jaerae	S(4)	S	S
136614	Keofalia Local	S(4)	R	S
136646	NPL-CGS-1986-22905	S(4)	S	S
158286	MI-221	S(4)	R	S
158287	MI-600	S(4)	S	S
158552	ANNABELLE	S(4)	S	S
203221	Mestnii	S(4)	S	S
218617	Mestnii	S(4)	R	S
264053	11PC7	S(4)	S	S
264054	11PC6	S(4)	S	S
264055	11PC5	S(4)	S	S
264056	11PC2	S(4)	R	S
267531	Largo de Reus	S(4)	S	S
267533	Maor	S(4)	S	S
267534	Najerano	S(4)	S	S
267536	Padron	S(4)	S	S
267541	Spain 1	S(4)	H	S
267543	Spain 12	S(4)	S	S
267545	Spain 9	S(4)	S	S
270400	12G542	S(4)	S	S
270425	AC12-176	S(4)	S	S
270426	AC12-197	S(3.5)	S	S
270434	Akashi-Piman	S(4)	R	S
270436	AC12-200	S(4)	S	S
270485	AC12-187	S(4)	S	S
270492	Chudai tian jiao wang	S(4)	S	S
270552	AC12-199	S(4)	S	S
270598	Long pin cai jiao jin yu	S(4)	S	S
270669	AC12-177	S(4)	S	S
270670	AC12-206	S(4)	R	S
270684	AC12-167	S(4)	S	S

R, resistant; H, heterozygous; S, susceptible.

These two markers were also applied to the 20 breeding lines (Hana Seed Co., Korea), in which the Phytophthora blight resistance was moderate or segregated (Table 6). Markers were analyzed for each line of 4–8 individuals. Among the 20 breeding lines, the 13 lines with moderate resistance showed the susceptibility allele for QTL5-1 and the resistance allele for Chr02-1126. As for the lines in which strong and weak resistance were segregating, either the marker locus had resistance or a heterozygous allele. Both P4435 and P4436, which demonstrated susceptibility, possessed susceptibility alleles for both markers.

**Table 6:** HRM analysis result of Chr02-1126 and QTL5-1 markers for breeding lines of Hana Seeds.

Sample ID	Expectation (Hana Co.)	Chr02-1126	QTL5-1	Disease severity score
P4180	Moderate	R	S	2.5
P4208	Moderate	R	S	2.5
P4236	Moderate	R	S	2.5
P4264	Moderate	R	S	2.5
P4292	Moderate	R	S	2.5
P4320	Moderate	R	S	2.5
P4348	Moderate	R	S	2.5
P4376	Moderate	R	S	2.5
P4404	Moderate	R	S	2.5
P4444	Moderate	R	S	2.5
P4463	Moderate	R	S	2.5
P4487	Moderate	R	S	2.5
P4518	Moderate	R	S	2.5
P4183	Segregating	H	R	1.5
P4189	Segregating	R	H	4
P4191	Segregating	R	S	4
P4192	Segregating	H	H	4
P4193	Segregating	R	S	4
P4435	Segregating	S	S	4
P4436	Segregating	S	S	4
CM334	Strong	R	R	0
Dokyachungchung	Strong	H	H	0
Manitta	Weak	S	S	4

R, resistant; H, heterozygous; S, susceptible.

## Discussion

*Phytophthora capsici* is the most devastating pathogen of pepper production around the world, and current management strategies are ineffective (Barchenger et al., 2018). Management aims to limit the losses associated with the pathogen because, once established, *P. capsici* is extremely difficult to eradicate (Lamour et al., 2011). Furthermore, *P. capsici* can easily disseminate from field to field and quickly establish itself in a given area, as the surface water used for irrigation is an important means of disseminating pathogens (Gevens et al., 2007). Since completely preventing *P. capsici* movement between sites is often unlikely, the best strategy for preventing *P. capsici* infection in vegetable crops is the development of resistant varieties, which is a less expensive and more viable alternative to fungicide use and other management techniques (Hausbeck and Lamour, 2004). Therefore, screening a wide range of genetic resources is critical for identifying resistant materials and applications for breeding purposes. We found only eight resistant accessions from *C. annuum* spp. and none from *C. baccatum*, *C. chacoense*, *C. chinense*, or *C. frutescens*. The chance of finding a resistant accession from *C. annuum* was high, which could be attributed to the fact that we used a large number of accessions from *C. annuum* spp. and a few from other species (Table 2).

Phenotypic data can be used to assess underlying variations in plant species and their responses to diseases. The GWAS is a method used in genetics research to link genetic variants to specific diseases or phenotypic traits. The introduction of many target enrichment or genome complexity reduction approaches, such as GBS, has made this form of GWAS practical for finding SNPs (Elshire et al., 2011; Glaubitz et al., 2014). In this study, a Phytophthora blight resistance evaluation with a KCP7 isolate in the pepper population (Lee et al., 2016) was performed using GBS and GWAS analysis. Through GWAS analysis, several significant SNPs associated with resistance to Phytophthora blight were identified. These SNPs were located on chromosomes Chr.01, Chr.02, Chr.03, Chr.04, Chr.06, Chr.07, Chr.08, and Chr.12. The most significantly associated SNP was located on Chr.04 (*p* = 2.47 × 10^−24^). The major effect QTLs for *P. capsici* root rot resistance was found on pepper chromosomes P5, P8, and P9. These QTLs were named QTL.Pc5, QTL.Pc8.1, and QTL.Pc9 (Lozada et al., 2021). Regardless of the resistance sources or *P. capsici* isolates, major resistance QTLs for *P. capsici* have been consistently detected in the close vicinity of chromosome P5 in several studies (Bonnet et al., 2007; Kim et al., 2008; Mallard et al., 2013; Liu et al., 2014; Rehrig et al., 2014). Based on a meta-analysis of QTLs, MetaPc5.1, the major effect QTL, was found to be located in the 22.4–24.6 Mb region on chromosome P5, while MetaPc5.2 and MetaPc5.3 were found to be located in the 53.0–162.6 Mb and 9.7–13.3 Mb regions on chromosome P5, respectively (Mallard et al., 2013; Wang et al., 2016). A number of minor and isolate-specific QTLs have been found, but their positions vary depending on the genetic background and the *P. capsici* isolate (Thabuis et al., 2004; Truong et al., 2012; Rehrig et al., 2014). Several molecular markers associated with key QTLs have been created and used for marker-assisted selection (MAS) in peppers, including Phyto5NBS1 and ZL6726. However, their application is limited to selecting resistant plants for extremely virulent and race-non-specific *P. capsici* isolates (Liu et al., 2014; Wang et al., 2016; Siddique et al., 2019). Eight QTLs with minor effects were detected on P2, P4, P8, and P11 against medium and highly aggressive isolates of *P. capsici* (Liu et al., 2014; Rehrig et al., 2014; Siddique et al., 2019). Since Phytophthora blight is resistant due to the actions of several genes, it is very difficult to select plants with markers from only one chromosomal locus. Therefore, it is very important not only to find the major resistance QTLs but also to find the minor resistance QTLs and develop a marker to evaluate them together. This is because bioassays for a lot of genetic resources require a lot of cost and effort.

Molecular markers, such as amplified fragment length polymorphisms (AFLPs), simple sequence repeats (SSRs), and, more commonly, single nucleotide polymorphisms, have been created and used in a variety of crops for different breeding programs (Kankanala et al., 2019). The availability of genome sequence data for a broad variety of plant species, including pepper, has made it simpler to employ molecular markers, such as SNP markers, to identify resistant resources for genetic improvement. One of the SNPs found on chromosome 2 was converted to an HRM marker (Chr02-1126). The QTL5-1 associated with the major QTL of *P. capsici* resistance (Liu et al., 2014; Siddique et al., 2019) and Chr02-1126 in this study were applied to 96 samples (Table 5) and 20 breeding lines (Table 6). Chr02-1126 could be used to determine the minor QTL for *P. capsici* resistance. In a previous study by Siddique et al. (2019), the minor resistance QTL was detected at 129–131 Mb on chromosome 2, and the SNP marker Chr02-1126 was located at 112 Mb on chromosome 2 (Table 4), demonstrating that the two QTLs might be the same minor QTL. One of the candidate genes for QTL on chromosome 2 was the WVD2-like gene. Plants with high constitutive expression of WVD2 or other members of the WVD2-LIKE (WDL) gene family have stems and roots that are short and thick, have reduced anisotropic cell elongation, and have inverted handedness of twisting in hypocotyls and roots, compared with wild types (Perrin et al., 2007). One SNP on chromosome 1 was found in a gene that encodes an NRT1/PTR FAMILY 7.3-like protein (Table 4).

On Chr.03, a significant SNP (at 26,843,710 bp) was discovered in a gene that encodes the TIME FOR COFFEE (TIC) protein (Table 4). Hong et al. (2014) revealed a previously unknown function of TIC in regulating root meristem growth in Arabidopsis through changes in auxin accumulation. Roots play a vital role in plant survival and productivity because they not only anchor the plants in the soil but are also the main organ for absorbing nutrients from the outside for better plant growth and development. Recent studies by Della Coletta et al. (2021) reported that a homolog of the Arabidopsis TIC gene is implicated in *Brachypodium distachyon*’s non-host resistance to wheat stem rust and also contributes to freezing tolerance in apples by promoting the unsaturation of fatty acids (Zhao et al., 2021). In fact, TIC, a part of Arabidopsis circadian light response gating, interacts with MYC2, resulting in proteasome-mediated degradation of the MYC2 protein. The *tic* mutant exhibits JA-associated phenotypes opposite to the myc2 mutant, such as increased susceptibility to *Pseudomonas syringae* pv. tomato (*Pst*) DC3000, JA sensitivity, and increased expression of the wound and insect-responsive genes VSP and TAT (tyrosine amino transferase), but decreased expression of the pathogen defense genes PDF1.2 and PR4 (Shin et al., 2012; Kazan and Manners, 2013). Another significant SNP that was found on Chr.03 (at 281,084,163 bp) encodes the F-box Kelch-repeat protein At3g06240-like. These previously reported findings, as well as the presence of the TCI gene in our study, may provide insight for future research into the role of the TCI gene in resistance to Phytophthora blight in *Capsicum* spp. Furthermore, a significant SNP was found on Chr.07 (at 205,297,202 bp) in the gene that encodes pentatricopeptide repeat (PPR) proteins (Table 4). Secondary phased siRNAs (phasiRNAs) were abundant in *Phytophthora soja*-infected soybean roots generated by genes producing nucleotide-binding domain, and leucine-rich repeat (NB-LRR) and PPR proteins, suggesting they may play a role in resistance to the disease (Wong et al., 2014). They also stated that the data indicated that specific miRNAs and phasiRNas regulate defense-associated genes in soybeans against *P. sojae* infection. The data suggest a link between decreased PPR gene expression and increased *P. sojae* resistance (Wong et al., 2014).

For more rapid selection, several molecular markers associated with resistance to *P. capsici* have been identified in peppers (Truong et al., 2012; Liu et al., 2014; Wang et al., 2016; Xu et al., 2016). However, these publicly available molecular markers have not been widely used, and when used in diverse germplasms, some phenotypic and genotypic mismatches have been observed (Barchenger et al., 2018). This phenotype–genotype mismatch limits the effectiveness of marker-assisted selection while emphasizing the pathogen’s high level of plasticity (Barchenger et al., 2018). The phenotype prediction accuracy of the markers used in this study was validated using 96 accessions and 20 breeding lines. The prediction accuracy for both markers was approximate. Marker Chr021126 had a prediction accuracy of 78.5%, while marker QTL5-1 had an accuracy of 80.2% (Figure 9). The QTL5-1 marker accurately predicted all of the susceptible plants (100%). When Chr02-1126 was applied to the susceptible plant, the prediction accuracy was 75%. These findings show that the QTL5-1 marker prediction accuracy with resistant pepper accessions was 70.3 percent, and the marker Chr02-1126 allowed to select resistant plants with a higher probability (78.1%).

## Conclusion

This study evaluated pepper accessions from five different *capsicum* spp. in an effort to identify resistant materials. Significant SNPs associated with Phytophthora blight resistance were found on different chromosomes. One SNP on Chr.02 was converted into an HRM marker (Chr02-1126) and used for validation in 96 pepper accessions and 20 breeding lines. The prediction accuracy of the Chr02-1126 marker for *P. capsici* was 78.5%. The Chr02-1126 marker, along with the major QTL marker, could help in the screening of resistance to Phytophthora blight for pepper genetic resources. The finding of SNPs associated with resistance and the selection of markers with greater prediction precision will help *Capsicum* breeding programs develop Phytophthora blight-resistant cultivars.

## Data Availability Statement

The data presented in the study are deposited in the NABIC repository, accession number NV-0728.

## Author Contributions

NR conducted the phenotyping in the core collection and HRM marker validation. JR, AH, BK, JL, and B-SH carried out disease assessment and severity scoring. BG and JL conducted HRM analysis. OH conducted genotyping in the core collection. MH wrote the manuscript and made the figures. B-CK supervised the overall processes and revised the manuscript. All authors contributed to the article and approved the submitted version.

## Funding

This work was carried out with the support of “The Cooperative Research Program for Agriculture Science and Technology Development (Project No. PJ013251022020)” National Institute of Agricultural Sciences, RDA, Republic of Korea.

## Conflict of Interest

The authors declare that the research was conducted in the absence of any commercial or financial relationships that could be construed as a potential conflict of interest.

## Publisher’s Note

All claims expressed in this article are solely those of the authors and do not necessarily represent those of their affiliated organizations, or those of the publisher, the editors and the reviewers. Any product that may be evaluated in this article, or claim that may be made by its manufacturer, is not guaranteed or endorsed by the publisher.

## Supplementary Material

The Supplementary Material for this article can be found online at: https://www.frontiersin.org/articles/10.3389/fpls.2022.902464/full#supplementary-material

## Footnotes

^1^ https://broadinstitute.github.io/picard/

^2^ http://pgd.pepper.snu.ac.kr

^3^ http://bioinfo.ut.ee/primer3/
