# Supplementary material for: Genome-Wide Association Study of Resistance to Phytophthora capsici in the Pepper (Capsicum spp.) Collection
Source: Front Plant Sci. 2022 May 20;13:902464. doi: 10.3389/fpls.2022.902464 (PMC9164128; doi:10.3389/fpls.2022.902464)
Supplement: Supplementary file 1 [file Data_Sheet_1.docx]

**Supplementary Data**

**Table S1.** Primer sequences used for HRM analysis

| Chr. | Position | Forward | Reverse |
| --- | --- | --- | --- |
| chr02 | 112632207 | GCCCCTTGCCTGCTATAACTA | CGAGGATGACATTTTCGTACC |
| chr02 | 115557669 | ACTTATTGTGAGCCTTGGGTAGAC | AAAATGTAAAATTGCAGCCACTTC |
| chr02 | 118473950 | TAAATTCAATGAGACAAGGGTGAT | CAGGTCGTTAAGAGAGTTGGACTT |
| chr02 | 165074534 | ACGGCTCGGTCCTCATCT | CGTATAACAACATCGAATCCATCG |
| chr02 | 165083237 | AATTATTATTGGTTCCCGATTGAA | TCTCGAGAAGTTCAAACAAAAATG |
| chr02 | 165083291 | TTACATTTTTGTTTGAACTTCTCG | AGTTGAGTACGATTATTTCCTTGTAGT |
| chr03 | 26843710 | CCATCTGTAAATTTTGCCTCGTAG | ATGGCACAGAATCATGCTATTTTT |
| chr03 | 203119513 | ACTCGCCACTATCTTTGGTAAAAC | TTTTGAGCCGTACTAATCATGAGA |
| chr03 | 273929454 | TGGACTGTTGAAAATTTGTCTGTC | TGCCTACTTTTAACCATTACTCCA |
| chr03 | 281374819 | AATTCAAATTTAACGCAGCTTAGG | GTTTAAGGAGGAATCAAATGTTGG |
| chr03 | 281374880 | CTTTTTCAGAACCCAACATTTGAT | ATTTACCTTGAGGATGACATAGGC |
| chr04 | 9578255 | GTAGGATCACTGACCAGTTATGGA | AACAATCGTGCTATGCATTACCTA |
| chr06 | 3204310 | CCCAAAAATGGTCCGTTCTC | AAGGCTGAGGAGTAGCCTAGTGT |
| chr06 | 184471306 | TCTGAAGTTCCACTAGATGACACC | CACCTTTACTTTCCCTTTTGAAGT |
| chr07 | 9144225 | CATTTCTGCCAGAGGTTGTTTC | GCCTTGGAGTAACTGGTAAAGTTG |
| chr07 | 205297202 | AGCTCATAGCATCTGGAATCTTGT | TAATATGCTGCTCAACAAGTCCTC |
| chr08 | 138930210 | CCATCTGGAGCAGATACAACTAGA | GGACACGCAGATTACGTTAAAAAT |
| chr09 | 3642467 | CGACGGTCTATTGTACACAGTCTT | ATGACTGCCATATGTGACAGGAG |
| chr09 | 16370729 | TTCGGACAGCTAAATTTGGATTAT | GTAGCTGCTGGAGAAGTCTTTGAG |
| chr12 | 232050702 | GGCTGCTATTTGGTCAAAGC | CGGTAGTCAATGCTCCAACTC |

**Table S2.** Disease severity scores of resistant and susceptible controls.

| **Control** | **Name** | **Disease severity score** | | | | |
| --- | --- | --- | --- | --- | --- | --- |
|  |  | **1^st^** | **2^nd^** | **3^rd^** | **Mean** | **STD.** |
| Resistant | CM334 | 0.0 | 0.0 | 0.0 | 0.0 | 0.0 |
|  | Dokyachungchung | 0.0 | 0.0 | 0.0 | 0.0 | 0.0 |
|  | Bigstar | 0.0 | 0.5 | 0.0 | 0.2 | 0.2 |
| Susceptible | Manitta | 4.0 | 3.9 | 4.0 | 4.0 | 0.0 |
|  | Cheungyang | 4.0 | 4.0 | 4.0 | 4.0 | 0.0 |
|  | Jeju-jaerae | 4.0 | 4.0 | 4.0 | 4.0 | 0.0 |

Note. STD: Standard deviation.

**Supplementary Figures**


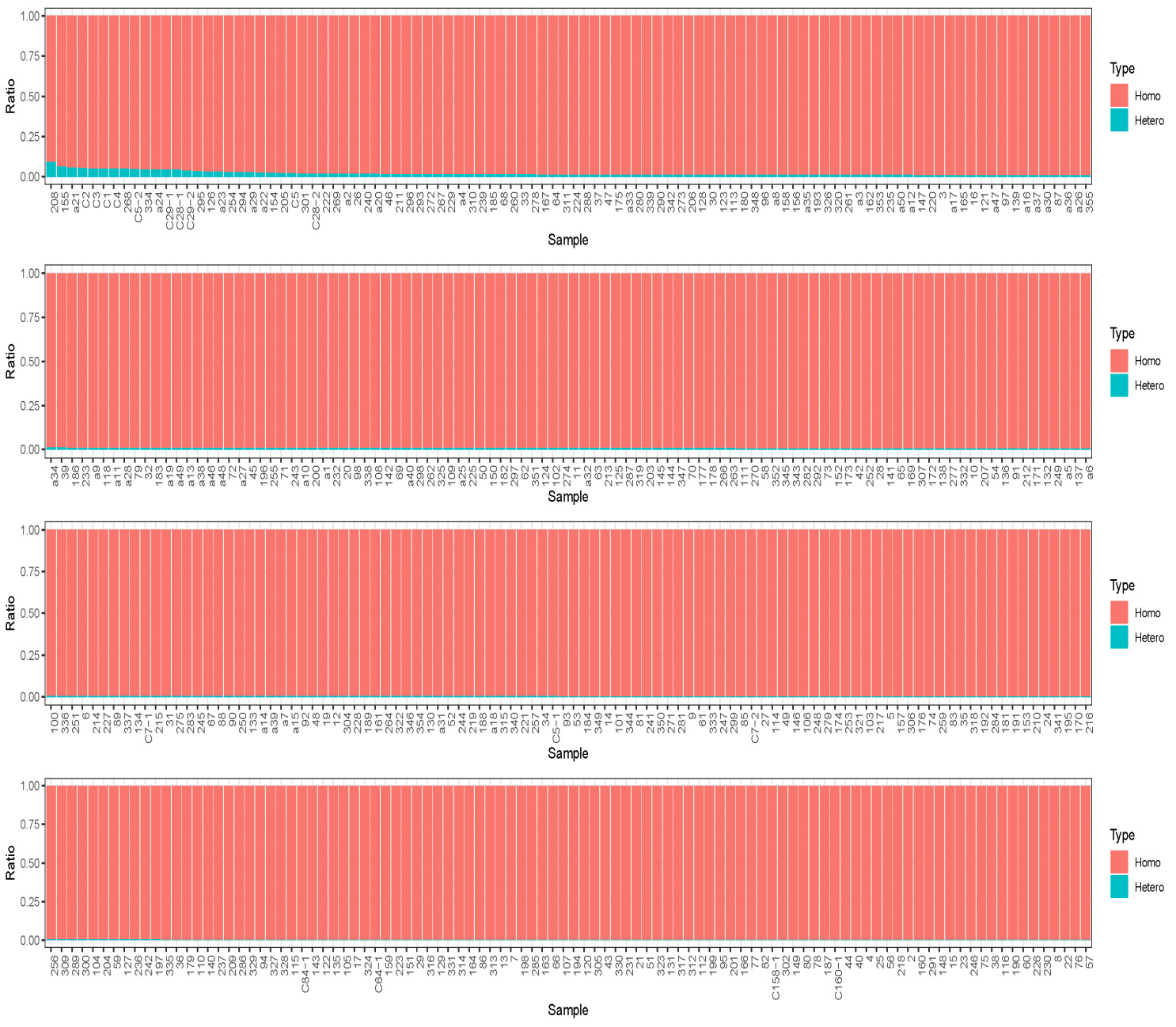


**Figure S1.** The ratio of homozygous or heterozygous loci of each accession. Red and green color indicate homozygous and heterozygous loci, respectively. Most of them are red, indicating that the most of plants in GWAS of pepper population have homozygous loci.


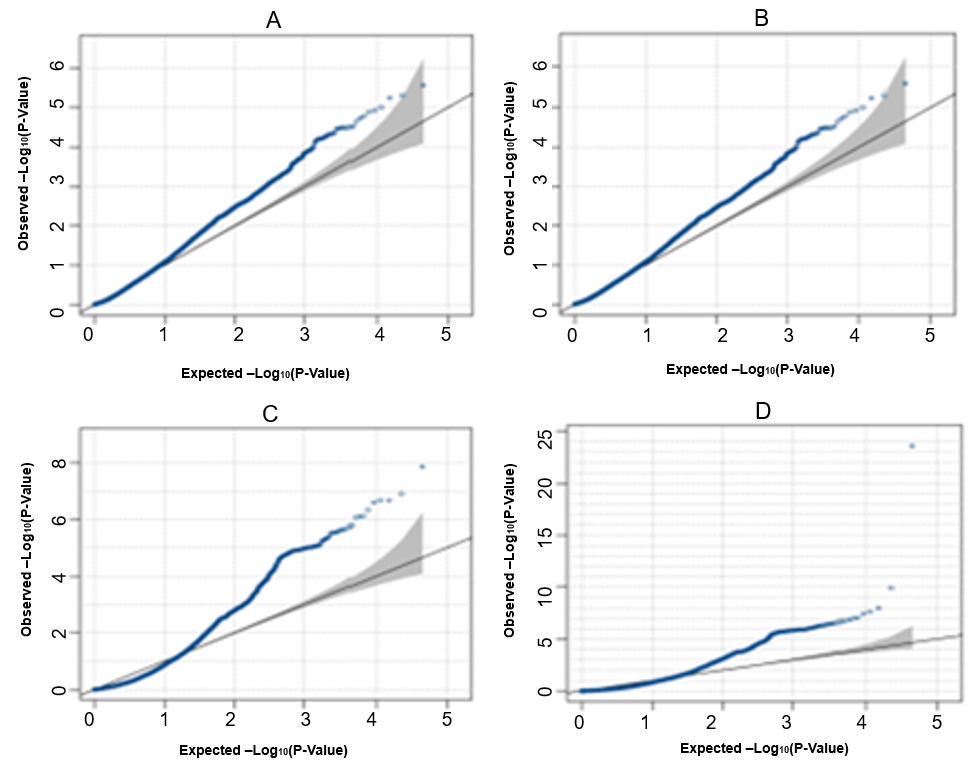


**Figure S2.** QQ plots of genome-wide association study on Phytophthora resistance in GWAS of pepper population. GWAS analysis was performed weekly to show the QQ plot.
